# Supplementary material for: Evolution of enhanced innate immune suppression by SARS-CoV-2 Omicron subvariants
Source: Nat Microbiol. 2024 Jan 16;9(2):451–63. doi: 10.1038/s41564-023-01588-4 (PMC10847042; doi:10.1038/s41564-023-01588-4)
Supplement: Supplementary file 1 — Reporting Summary [file 41564_2023_1588_MOESM1_ESM.pdf]

nature portfolio

Corresponding author(s): Ann-Kathrin Reuschl
Clare Jolly
Greg. J. Towers

Last updated by author(s): Nov 27, 2023

Reporting Summary

Nature Portfolio wishes to improve the reproducibility of the work that we publish. This form provides structure for consistency and transparency in reporting. For further information on Nature Portfolio policies, see our Editorial Policies and the Editorial Policy Checklist.

Statistics

For all statistical analyses, confirm that the following items are present in the figure legend, table legend, main text, or Methods section.

n/a	Confirmed	
☐	☒	The exact sample size (*n*) for each experimental group/condition, given as a discrete number and unit of measurement
☐	☒	A statement on whether measurements were taken from distinct samples or whether the same sample was measured repeatedly
☐	☒	The statistical test(s) used AND whether they are one- or two-sided<br>*Only common tests should be described solely by name; describe more complex techniques in the Methods section.*
☐	☒	A description of all covariates tested
☐	☒	A description of any assumptions or corrections, such as tests of normality and adjustment for multiple comparisons
☐	☒	A full description of the statistical parameters including central tendency (e.g. means) or other basic estimates (e.g. regression coefficient) AND variation (e.g. standard deviation) or associated estimates of uncertainty (e.g. confidence intervals)
☐	☒	For null hypothesis testing, the test statistic (e.g. *F*, *t*, *r*) with confidence intervals, effect sizes, degrees of freedom and *P* value noted<br>*Give P values as exact values whenever suitable.*
☒	☐	For Bayesian analysis, information on the choice of priors and Markov chain Monte Carlo settings
☒	☐	For hierarchical and complex designs, identification of the appropriate level for tests and full reporting of outcomes
☒	☐	Estimates of effect sizes (e.g. Cohen's *d*, Pearson's *r*), indicating how they were calculated

Our web collection on statistics for biologists contains articles on many of the points above.

Software and code

Policy information about availability of computer code

Data collection	BD FACSDiva Software Verison 9.0 (BD), Odyssey Infrared Imager software Image Studia Lite V5.2, 7500 Software (Applied Biosystems), WiScan® Hermes 7-Colour High-Content Imaging System (IDEA Bio-Medical).
Data analysis	FlowJo v10.6.2 (Tree Star); Design & Analysis Software Version: 2.6.2 (Thermo Fisher Scientific);Image Studia Lite V5.2 (LI-COR); GraphPad Prism9 Versino 9.0.0; FIJI ImageJ software package40; Athena Image analysis software (IDEA Bio-Medical)

For manuscripts utilizing custom algorithms or software that are central to the research but not yet described in published literature, software must be made available to editors and reviewers. We strongly encourage code deposition in a community repository (e.g. GitHub). See the Nature Portfolio guidelines for submitting code & software for further information.

Data

Policy information about availability of data

All manuscripts must include a data availability statement. This statement should provide the following information, where applicable:

- Accession codes, unique identifiers, or web links for publicly available datasets
- A description of any restrictions on data availability
- For clinical datasets or third party data, please ensure that the statement adheres to our policy

All data generated or analysed during this study are included in this manuscript (and its supplementary information files). SARS-CoV-2 sequence counts were extracted from CoV-Spectrum (cov-spectrum.org) using genomic data from GSAID. No new algorithms were developed for this project.

Human research participants

Policy information about studies involving human research participants and Sex and Gender in Research.

Reporting on sex and gender	Not applicable.
Population characteristics	Not applicable.
Recruitment	Not applicable.
Ethics oversight	Not applicable.

Note that full information on the approval of the study protocol must also be provided in the manuscript.

Field-specific reporting

Please select the one below that is the best fit for your research. If you are not sure, read the appropriate sections before making your selection.

☒ Life sciences ☐ Behavioural & social sciences ☐ Ecological, evolutionary & environmental sciences

For a reference copy of the document with all sections, see nature.com/documents/nr-reporting-summary-flat.pdf

Life sciences study design

All studies must disclose on these points even when the disclosure is negative.

Sample size	No statistical methods were used to pre-determine sample sizes but our sample sizes are similar to those reported in previous publications (Thorne et al, 2022, Nature; Thorne et al, 2021, EMBO J). Multiple independent experiments were repeated to allow for appropriate statistical analysis.
Data exclusions	No data were excluded.
Replication	In vitro experiments were performed independently a minimum of 3 times (unless otherwise stated) to allow for appropriate confidence. All attempts at replication were successful.
Randomization	No randomisation was performed. Experimental groups were treated identical except for the specific variable being tested, thus randomisation is not required.
Blinding	Blinding was not necessary all measurements were quantified by automated machines, and no data were excluded.

Reporting for specific materials, systems and methods

We require information from authors about some types of materials, experimental systems and methods used in many studies. Here, indicate whether each material, system or method listed is relevant to your study. If you are not sure if a list item applies to your research, read the appropriate section before selecting a response.

Materials & experimental systems

n/a	Involved in the study	
☐	☒	Antibodies
☐	☒	Eukaryotic cell lines
☒	☐	Palaeontology and archaeology
☒	☐	Animals and other organisms
☒	☐	Clinical data
☒	☐	Dual use research of concern

Methods

n/a	Involved in the study	
☒	☐	ChIP-seq
☐	☒	Flow cytometry
☒	☐	MRI-based neuroimaging

Antibodies

Antibodies used	For flowcytometry and western blot:<br>CR3009 SARS-CoV-2 cross-reactive antibody (a gift from Laura McCoy); secondary Alexa Fluor 488-Donkey-anti-Human IgG (Jackson Labs); rabbit-anti-SARS spike (Invitrogen, PA1-411-1165), mouse-anti-SARS-CoV-2 spike (GeneTex 1A9), rabbit-anti-Orf6 (Abnova, PAB31757), rabbit-anti-Orf9b (ProSci, 9191), rabbit-anti-phospho-STAT1 (Ser727) (CellSignaling, Cat # 9177), rabbit-anti-phospho

STAT1 (Tyr 701) (CellSignaling, Cat# 9167, clone 58D6), rabbit-anti-STAT1 (CellSignaling, Cat# 9172), anit-rabbit-IRF3 (CellSignaling, Cat# 4302), rabbit-anti-phospho IRF3 (CellSignaling, Cat# 29047, clone D6O1M) and rabbit-anti-beta-actin (A2066, SIGMA), IRDye 800CW or 680RD secondary antibodies (Abcam, goat anti-rabbit, goat anti-mouse or goat anti-human).
For immunofluorescence microscopy:
Rabbit-anti-IRF3 antibody (sc-33641, Santa Cruz), rabbit-anti-STAT-1 (D1K9Y, Abcam), mouse-anti-dsRNA (MABE1134, Millipore) and Cr3009 SARS-CoV cross-reactive human-anti-N antibodies; anti-rabbit-AlexaFluor-488. anti-mouse-AlexaFluor-568 and anti-human-Alexa647 conjugates (Jackson ImmunoResearch).

Validation

Validation for commercial antibodies and target specificity was confirmed in the technical data sheets provided by the manufacturer, containing example data and relevant citations. Furthermore, negative controls (uninfected cells) were included in experiments to confirm absence of or low non-specific binding of antibodies.

CR3009 SARS-CoV-2 cross-reactive: described and validated here https://www.nibsc.org/documents/ifu/101009.pdf
rabbit-anti-SARS spike (Invitrogen, PA1-411-1165), validated by the manufacturer: https://www.thermofisher.com/antibody/product/SARS-Coronavirus-Spike-Protein-Antibody-Polyclonal/PA1-41165
mouse-anti-SARS-CoV-2 spike (GeneTex 1A9), validated by the manucfacturer. This antibody detects both SARS-CoV spike and SARS-CoV-2 spike proteins (S2 subunit). Based on sequence analysis, this antibody is predicted to recognize S2' subunit. Our internal testing indicates no cross-reactivity with MERS-CoV spike protein.This antibody is able to detect multiple SARS-CoV-2 VOCs, including Omicron variant. https://www.genetex.com/Product/Detail/SARS-CoV-SARS-CoV-2-COVID-19-spike-antibody-1A9/GTX632604
rabbit-anti-Orf6 (Abnova, PAB31757), details in the technical datasheet: https://www.abnova.com/upload/media/product/document/2020/DS_PAB31757.pdf
rabbit-anti-Orf9b (ProSci, 9191), details provided by the manufacturer: https://www.prosci-inc.com/product/sars-cov-2-covid-19-orf9b-antibody-9191/
rabbit-anti-phospho-STAT1 (Ser727) (CellSignaling, Cat # 9177), Phospho-Stat1 (Ser727) Antibody detects endogenous levels of Stat1α only when phosphorylated at Ser727. This site is deleted in Stat1β. This antibody does not significantly cross-react with the corresponding phosphorylated residues of other Stat proteins. https://www.cellsignal.com/products/primary-antibodies/phospho-stat1-ser727-antibody/9177
rabbit-anti-phospho STAT1 (Tyr 701) (CellSignaling, Cat# 9167, clone 58D6), Phospho-Stat1 (Tyr701) (58D6) Rabbit mAb detects endogenous levels of Stat1 only when phosphorylated at tyrosine 701. The antibody detects phosphorylated tyrosine 701 of p91 Stat1 and also the p84 splice variant. It does not cross-react with the corresponding phospho-tyrosines of other Stat proteins. https://www.cellsignal.com/products/primary-antibodies/phospho-stat1-tyr701-58d6-rabbit-mab/9167
rabbit-anti-STAT1 (CellSignaling, Cat# 9172), Stat1 Antibody detects endogenous levels of total Stat1 protein. The antibody detects both Stat1alpha (91kDa) and Stat1beta (84 kDa) isoforms. https://www.cellsignal.com/products/primary-antibodies/stat1-antibody/9172
anit-rabbit-IRF3 (CellSignaling, Cat# 4302), IRF-3 (D83B9) Rabbit mAb detects endogenous levels of total IRF-3 protein. https://www.cellsignal.com/products/primary-antibodies/irf-3-d83b9-rabbit-mab/4302
rabbit-anti-phospho IRF3 (CellSignaling, Cat# 29047, clone D6O1M); Phospho-IRF-3 (Ser396) (D6O1M) Rabbit mAb recognizes endogenous levels of IRF-3 protein only when phosphorylated at Ser396. https://www.cellsignal.com/products/primary-antibodies/phospho-irf-3-ser396-d6o1m-rabbit-mab/29047
rabbit-anti-beta-actin (A2066, SIGMA), validation and details provided by the manufacturer: https://www.sigmaaldrich.com/GB/en/product/sigma/a2066
Rabbit-anti-IRF3 antibody (sc-33641, Santa Cruz), IRF-3 (SL-12) is recommended for detection of IRF-3 of mouse, rat and human origin. https://datasheets.scbt.com/sc-33641.pdf
rabbit-anti-STAT-1 (CellSignaling, Cat# 14994, clone D1K9Y), Stat1 (D1K9Y) Rabbit mAb recognizes endogenous levels of total Stat1 protein. This antibody also cross-reacts with an unidentified protein of 150 kDa. https://www.cellsignal.com/products/primary-antibodies/stat1-d1k9y-rabbit-mab/14994
mouse-anti-dsRNA (MABE1134, Millipore), Clone rJ2 specifically recognizes double stranded RNA (dsRNA) of greater than 40 bp in length that is generated during the replication of positive sense genome viruses. https://www.merckmillipore.com/GB/en/product/Anti-dsRNA-Antibody-clone-rJ2,MM_NF-MABE1134-25UL

Eukaryotic cell lines

Policy information about cell lines and Sex and Gender in Research

Cell line source(s)

Calu-3 cells were purchased from AddexBio (C0016001), Caco-2 cells were a kind gift from Dalan Bailey (Pirbright Institute) and Hela-ACE2 cells were a gift from James E Voss described in Rogers et al, 2020, Science. A459 cells expressing ACE2 and TMPRSS2 were a gift from Massimo Palmarini (CVR, Glasgow) described in Willet et al., 2022, Nature Microbiology.

Authentication

Cell lines were commercially procured and confirmed to be the cell lines indicated and mycoplasma-free by the supplier. Cell lines that were received from collaborators were confirmed to be the cell lines indicated by the respective labs.

Mycoplasma contamination

Random mycoplasma testing was conducted and cells tested negative.

Commonly misidentified lines
(See ICLAC register)

None

Flow Cytometry

Plots

Confirm that:

☒ The axis labels state the marker and fluorochrome used (e.g. CD4-FITC).

☒ The axis scales are clearly visible. Include numbers along axes only for bottom left plot of group (a 'group' is an analysis of identical markers).

☒ All plots are contour plots with outliers or pseudocolor plots.

☒ A numerical value for number of cells or percentage (with statistics) is provided.

Methodology

Sample preparation	Adherent cells were trypsinised and fixed in 4% formaldehyde prior to intracellular staining for SARS-CoV-2 nucleocapsid (N) protein. For N detection, cells were permeabilised for 15 min with Intracellular Staining Perm Wash Buffer (BioLegend) and subsequently incubated with 1μg/ml CR3009 SARS-CoV-2 cross-reactive antibody (a gift from Laura McCoy) for 30 min at room temperature. Primary antibodies were detected by incubation with secondary Alexa Fluor 488-Donkey-anti-Human IgG (Jackson Labs).
Instrument	All samples were acquired on a BD Fortessa X20 or LSR II using BD FACSDiva software.
Software	Data was analysed using FlowJo v10 (Tree Star).
Cell population abundance	N/A. Flowcytometry was used to determine SARS-CoV-2 infection levels in Calu-3 monocultures.
Gating strategy	Infected cells were identified as follows: Calu-3 cells identified by SSC-A vs FSC-A -> Singlets (FSC-A vs FSC-H) -> Nucleocapsid positive cells (N+) (gated on uninfected cells)

☒ Tick this box to confirm that a figure exemplifying the gating strategy is provided in the Supplementary Information.
