# Supplementary material for: Evolution of enhanced innate immune suppression by SARS-CoV-2 Omicron subvariants
Source: Nat Microbiol. 2024 Jan 16;9(2):451–63. doi: 10.1038/s41564-023-01588-4 (PMC10847042; doi:10.1038/s41564-023-01588-4)

Figure 2a

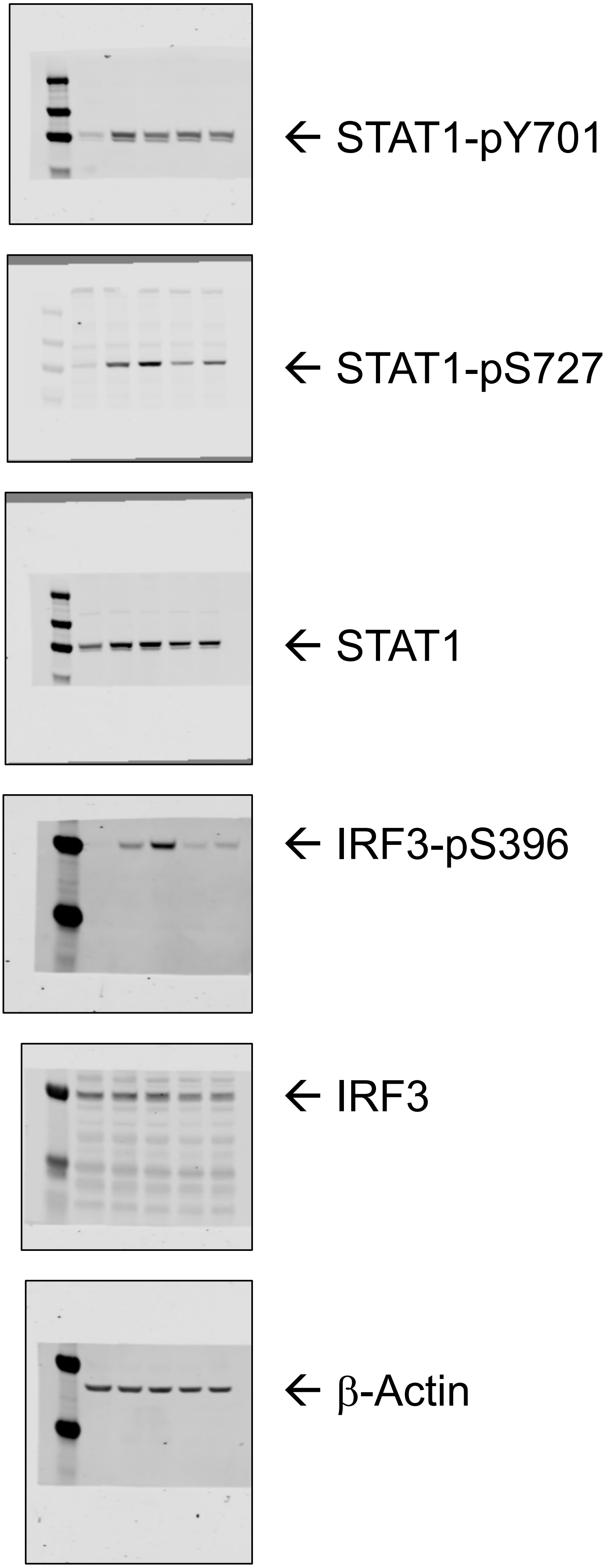

Figure 2h

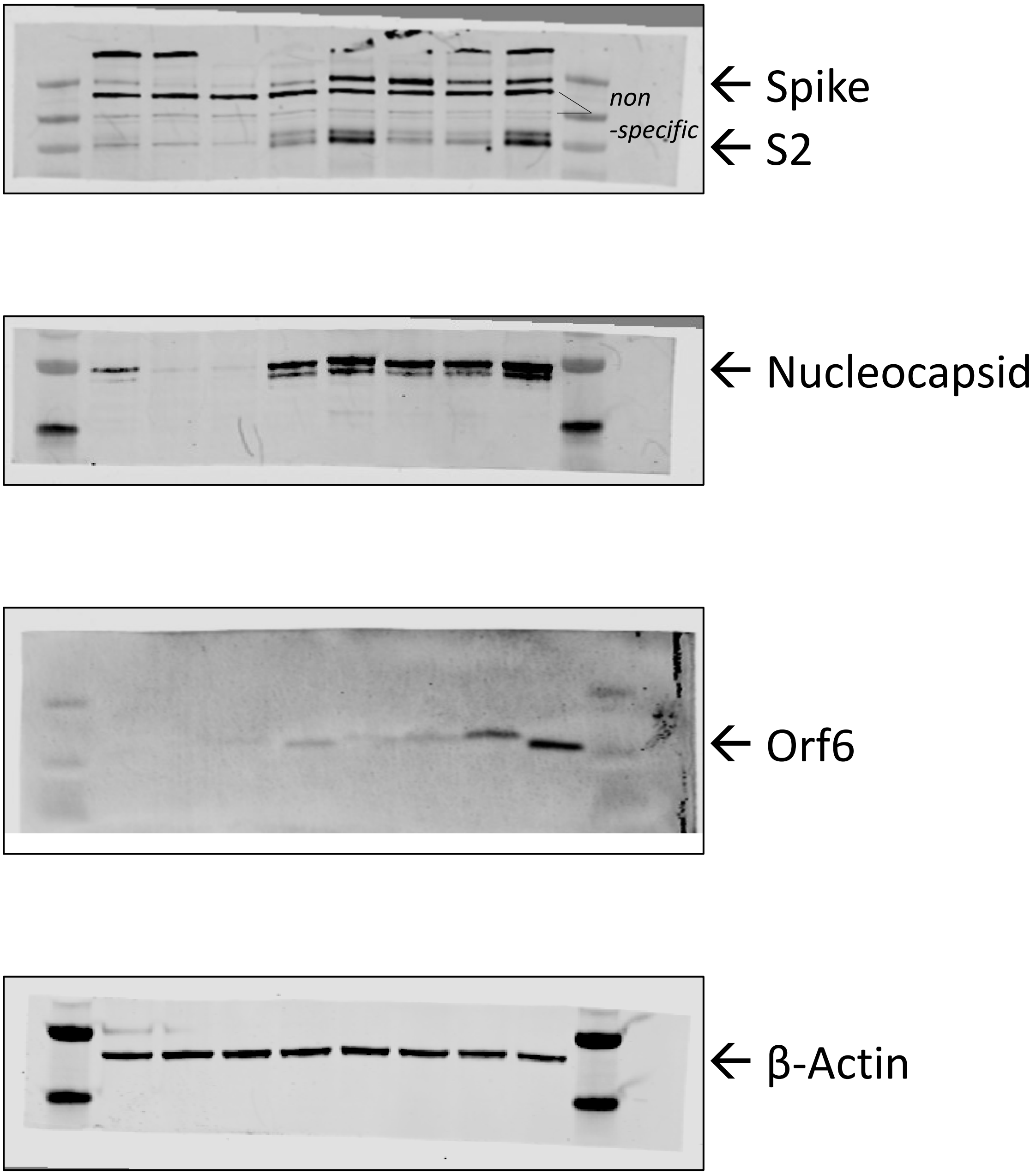

Figure 2n

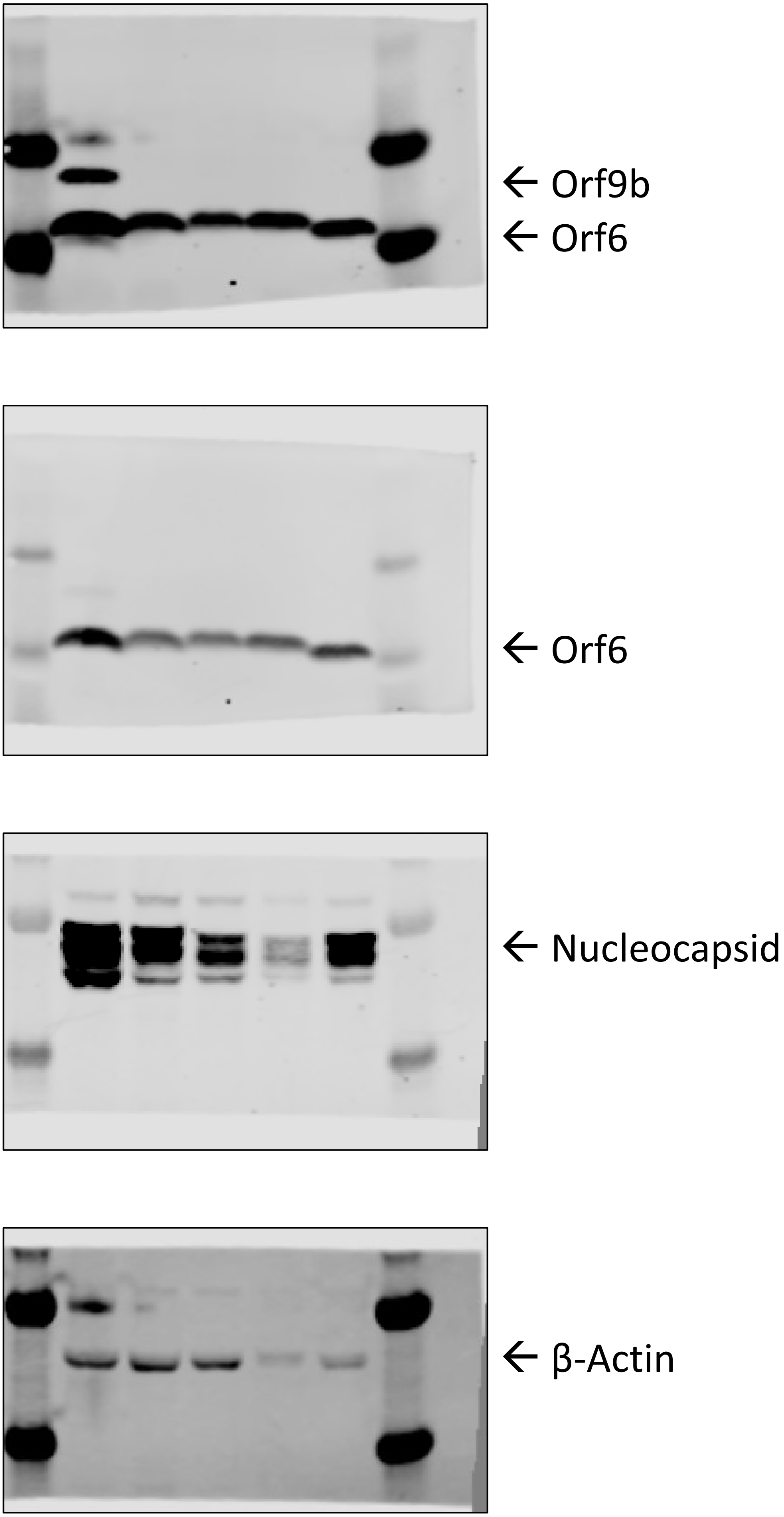

Supplement: Supplementary file 4 — Unprocessed western blots for Fig. 2. [file 41564_2023_1588_MOESM4_ESM.pdf]
